# Supplementary material for: Whole-genome sequencing reveals origin and evolution of influenza A(H1N1)pdm09 viruses in Lincang, China, from 2014 to 2018
Source: PLoS One. 2020 Jun 24;15(6):e0234869. doi: 10.1371/journal.pone.0234869 (PMC7314029; doi:10.1371/journal.pone.0234869)
Supplement: S1 Table — N/A: not applied. (DOC) [file pone.0234869.s001.doc]

**S1 Table. Accession numbers in GISAID databases of applied gene segments of 14 A(H1N1)pdm09 reference strains included in the analysis.** N/A: not applied.

| **Reference Strains** | **HA** | **NA** | **M** | **NP** | **NS** | **PA** | **PB1** | **PB2** |
| --- | --- | --- | --- | --- | --- | --- | --- | --- |
| A/California/7/2009 | EPI177294 | EPI185379 | EPI176619 | EPI184298 | EPI176618 | EPI176617 | EPI176615 | EPI176616 |
| A/Michigan/45/2015 | EPI662594 | EPI662593 | EPI662589 | EPI662587 | EPI662588 | EPI662590 | EPI662592 | EPI662591 |
| A/Brisbane/02/2018 | EPI1312566 | EPI1312565 | EPI1312561 | EPI1312559 | EPI1312560 | EPI1312562 | EPI1312564 | EPI1312563 |
| A/Osaka/1/2009 | EPI180717 | EPI180733 | N/A | | | | | |
| A/Lviv/N6/2009 | EPI239666 | EPI239665 | N/A | | | | | |
| A/Astrakhan/1/2011 | EPI319590 | EPI319591 | N/A | | | | | |
| A/Brisbane/10/2010 | EPI745532 | EPI745533 | N/A | | | | | |
| A/St. Petersburg/100/2011 | EPI316435 | EPI316434 | N/A | | | | | |
| A/St. Petersburg/27/2011 | EPI319527 | EPI319528 | N/A | | | | | |
| A/Hong Kong/5659/2012 | EPI382424 | EPI382425 | N/A | | | | | |
| A/Dakar/02/2014 | EPI539470 | EPI539471 | N/A | | | | | |
| A/South Africa/3626/2013 | EPI577031 | EPI577030 | EPI614494 | EPI614492 | EPI614493 | EPI614495 | EPI614497 | EPI614496 |
| A/Iowa/53/2015 | EPI676441 | EPI676440 | EPI676436 | EPI676434 | EPI676435 | EPI676437 | EPI676439 | EPI676438 |
| A/Victoria/55/2017 | EPI1082690 | EPI1082689 | N/A | | | | | |
