# Supplementary material for: Whole-genome sequencing reveals origin and evolution of influenza A(H1N1)pdm09 viruses in Lincang, China, from 2014 to 2018
Source: PLoS One. 2020 Jun 24;15(6):e0234869. doi: 10.1371/journal.pone.0234869 (PMC7314029; doi:10.1371/journal.pone.0234869)
Supplement: S2 Table — (DOC) [file pone.0234869.s002.doc]

**S2 Table. The number (%) of influenza strains isolated from samples of influenza-like cases in Lincang, China, from 2014 to 2018.**

|  | | **Number (%) of isolated strains** | | |
| --- | --- | --- | --- | --- |
| **Year** | **Number of**  **samples tested** | **Type A** | **Type B** | **Total(each year)** |
| 2014 | 1248 | 25(2.00) | 9(0.72) | 34(2.72) |
| 2015 | 1322 | 16(1.21) | 41(3.10) | 57(4.31) |
| 2016 | 1544 | 16(1.04) | 37(2.40) | 53(3.43) |
| 2017 | 1385 | 85(6.14) | 22(1.59) | 107(7.73) |
| 2018 | 1268 | 64(5.05) | 77(6.07) | 141(11.12) |
| Total(all years) | 6767 | 206(3.04) | 186(2.75) | 392(5.79) |
