# Supplementary material for: Whole-genome sequencing reveals origin and evolution of influenza A(H1N1)pdm09 viruses in Lincang, China, from 2014 to 2018
Source: PLoS One. 2020 Jun 24;15(6):e0234869. doi: 10.1371/journal.pone.0234869 (PMC7314029; doi:10.1371/journal.pone.0234869)
Supplement: S3 Table — (DOC) [file pone.0234869.s003.doc]

**S3 Table. Demographic characteristics of the cases infected with influenza viruses in Lincang, China, from 2014 to 2018.**

| **Classification** | **Number(%) of the cases infected with influenza viruses** | | | | | |
| --- | --- | --- | --- | --- | --- | --- |
| **Year(Total cases)** | 2014(34) | 2015(57) | 2016(53) | 2017(107) | 2018(141) | Total(392) |
| **Sex** |  |  |  |  |  |  |
| female | 6(17.65) | 18(31.58) | 27(50.94) | 39(36.45) | 60(42.55) | 150(38.27) |
| male | 28(82.35) | 39(68.42) | 26(49.06) | 68(63.55) | 81(57.45) | 242(61.73) |
| **Age group(years)** |  |  |  |  |  |  |
| 0～ | 25(73.53) | 26(45.61) | 30(56.60) | 60(56.07) | 78(55.32) | 219(55.87) |
| 5～ | 9(26.47) | 20(35.09) | 14(26.42) | 29(27.10) | 46(32.62) | 118(30.10) |
| 15～ | 0(0.00) | 8(14.04) | 4(7.55) | 6(5.61) | 7(4.96) | 25(6.38) |
| 25～ | 0(0.00) | 3(5.26) | 5(9.43) | 9(8.41) | 9(6.38) | 26(6.63) |
| 60～ | 0(0.00) | 0(0.00) | 0(0.00) | 3(2.80) | 1(0.71) | 4(1.02) |
| **Occupation** |  |  |  |  |  |  |
| Nursery children | 10(29.41) | 14(24.56) | 12(22.64) | 28(26.17) | 60(42.55) | 124(31.63) |
| Scattered children | 18(52.94) | 21(36.84) | 24(45.28) | 46(42.99) | 45(31.91) | 154(39.29) |
| Students | 6(17.65) | 16(28.07) | 9(16.98) | 19(17.76) | 27(19.15) | 77(19.64) |
| Cadre and employees | 0(0.00) | 2(3.51) | 1(1.89) | 1(0.93) | 2(1.42) | 6(1.53) |
| Workers | 0(0.00) | 3(5.26) | 6(11.32) | 2(1.87) | 6(4.26) | 17(4.33) |
| Farmers | 0(0.00) | 1(1.75) | 1(1.89) | 11(10.28) | 0(0.00) | 13(3.32) |
| Others | 0(0.00) | 0(0.00) | 0(0.00) | 0(0.00) | 1(0.71) | 1(0.26) |
