# Supplementary material for: Whole-genome sequencing reveals origin and evolution of influenza A(H1N1)pdm09 viruses in Lincang, China, from 2014 to 2018
Source: PLoS One. 2020 Jun 24;15(6):e0234869. doi: 10.1371/journal.pone.0234869 (PMC7314029; doi:10.1371/journal.pone.0234869)
Supplement: S5 Table — The evolutionary rates were estimated by BEAST v1.10.4 software under the uncorrelated relaxed clock model. (DOC) [file pone.0234869.s005.doc]

**S5 Table. Estimated evolutionary rates of HA genes of influenza A(H1N1)pdm09 strains in Lincang, China, from 2014 to 2018.** The evolutionary rates were estimated by BEAST v1.10.4 software under the uncorrelated relaxed clock model.

| **Strain No.** | **Evolutionary Rate (Substitutions/Site/Year)** | **95%HPD** |
| --- | --- | --- |
| A/Yunnan-Linxiang/SWL116/2018 | 0.0044 | [0.0009,0.009] |
| A/Yunnan-Linxiang/SWL1766/2018 | 0.0031 | [0.0006,0.0069] |
| A/Yunnan-Linxiang/SWL1765/2018 | 0.005 | [0.0011,0.0111] |
| A/Yunnan-Linxiang/SWL115/2018 | 0.0024 | [0,0.0075] |
| A/Yunnan-Linxiang/SWL1288/2018 | 0.0021 | [0,0.0066] |
| A/Yunnan-Linxiang/SWL1666/2018 | 0.0034 | [0.0001,0.0098] |
| A/Yunnan-Linxiang/SWL1586/2018 | 0.002 | [0,0.0063] |
| A/Yunnan-Linxiang/SWL1763/2018 | 0.0027 | [0.0001,0.0076] |
| A/Yunnan-Linxiang/SWL1715/2018 | 0.0018 | [0,0.0054] |
| A/Yunnan-Linxiang/SWL1708/2018 | 0.0028 | [0.0001,0.0076] |
| A/Yunnan-Linxiang/SWL119/2018 | 0.0083 | [0.0002,0.0234] |
| A/Yunnan-Linxiang/SWL118/2018 | 0.0118 | [0.0005,0.0332] |
| A/Yunnan-Linxiang/SWL1762/2018 | 0.0039 | [0.0008,0.0076] |
| A/Yunnan-Linxiang/SWL1863/2016 | 0.0116 | [0.0011,0.0313] |
| A/Yunnan-Linxiang/SWL333/2017 | 0.0029 | [0,0.0093] |
| A/Yunnan-Linxiang/SWL1895/2017 | 0.0013 | [0,0.0038] |
| A/Yunnan-Linxiang/SWL11043/2017 | 0.001 | [0,0.0027] |
| A/Yunnan-Linxiang/SWL1615/2017 | 0.0054 | [0,0.0163] |
| A/Yunnan-Linxiang/SWL332/2017 | 0.0043 | [0.0001,0.0133] |
| A/Yunnan-Linxiang/SWL326/2017 | 0.0044 | [0,0.0132] |
| A/Yunnan-Linxiang/SWL327/2017 | 0.0024 | [0,0.0076] |
| A/Yunnan-Linxiang/SWL1614/2017 | 0.0039 | [0,0.0113] |
| A/Yunnan-Linxiang/SWL324/2017 | 0.0043 | [0,0.0143] |
| A/Yunnan-Linxiang/SWL329/2017 | 0.0023 | [0,0.0073] |
| A/Yunnan-Linxiang/SWL1603/2017 | 0.0021 | [0,0.0066] |
| A/Yunnan-Linxiang/SWL1618/2017 | 0.002 | [0,0.006] |
| A/Yunnan-Linxiang/SWL330/2017 | 0.0023 | [0,0.0077] |
| A/Yunnan-Linxiang/SWL1505/2017 | 0.0028 | [0,0.009] |
| A/Yunnan-Linxiang/SWL331/2017 | 0.0085 | [0.0003,0.0251] |
| A/Yunnan-Linxiang/SWL1609/2017 | 0.0112 | [0.0008,0.033] |
| A/Yunnan-Linxiang/SWL1768/2018 | 0.0017 | [0.0004,0.0033] |
| A/Yunnan-Linxiang/SWL1865/2016 | 0.0028 | [0,0.0079] |
| A/Yunnan-Linxiang/SWL1612/2017 | 0.0087 | [0.0004,0.0263] |
| A/Yunnan-Linxiang/SWL1620/2017 | 0.0076 | [0.0003,0.0223] |
| A/Yunnan-Linxiang/SWL1621/2017 | 0.0021 | [0,0.0068] |
| A/Yunnan-Linxiang/SWL1894/2017 | 0.0012 | [0,0.0033] |
| A/Yunnan-Linxiang/SWL1703/2016 | 0.0026 | [0,0.0083] |
| A/Yunnan-Linxiang/SWL1171/2014 | 0.0022 | [0,0.007] |
| A/Yunnan-Linxiang/SWL1541/2016 | 0.0022 | [0,0.0073] |
| A/Yunnan-Linxiang/SWL1724/2016 | 0.0019 | [0,0.0059] |
| A/Yunnan-Linxiang/SWL1704/2016 | 0.002 | [0,0.006] |
| A/Yunnan-Linxiang/SWL1712/2016 | 0.0008 | [0,0.0021] |
| A/Yunnan-Linxiang/SWL1177/2014 | 0.0023 | [0,0.0073] |
| A/Yunnan-Linxiang/SWL1170/2014 | 0.0024 | [0,0.0077] |
| A/Yunnan-Linxiang/SWL1180/2014 | 0.0024 | [0,0.0076] |
