# Supplementary figures and images for: Whole-genome sequencing reveals origin and evolution of influenza A(H1N1)pdm09 viruses in Lincang, China, from 2014 to 2018
Source: PLoS One. 2020 Jun 24;15(6):e0234869. doi: 10.1371/journal.pone.0234869 (PMC7314029; doi:10.1371/journal.pone.0234869)

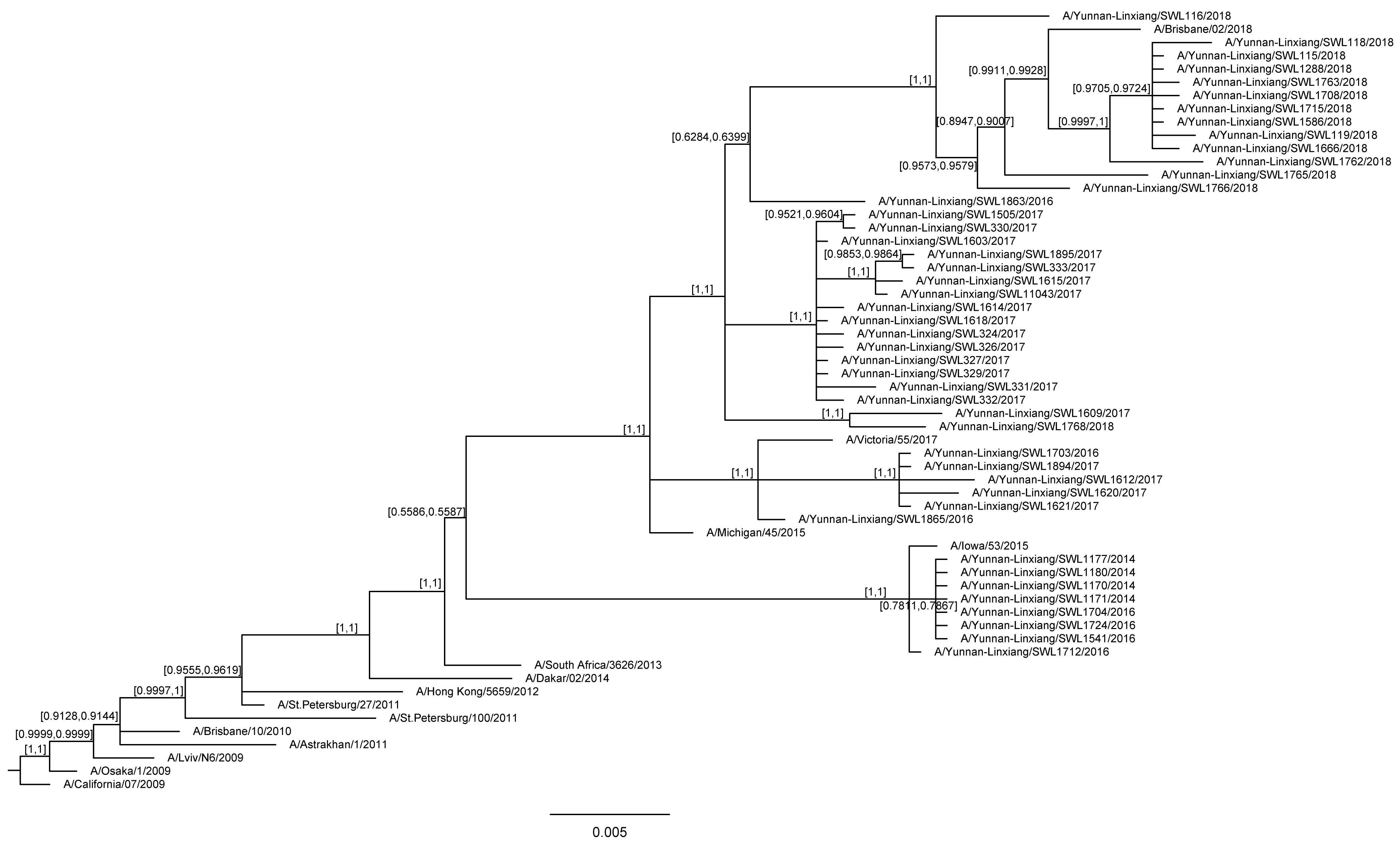

Supplement: S1 Fig — Each nodal number in square brackets represented a Bayesian posterior probability (BPP) range. The ruler value (0.005) represented genetic distance. (TIF) [file pone.0234869.s008.tif]

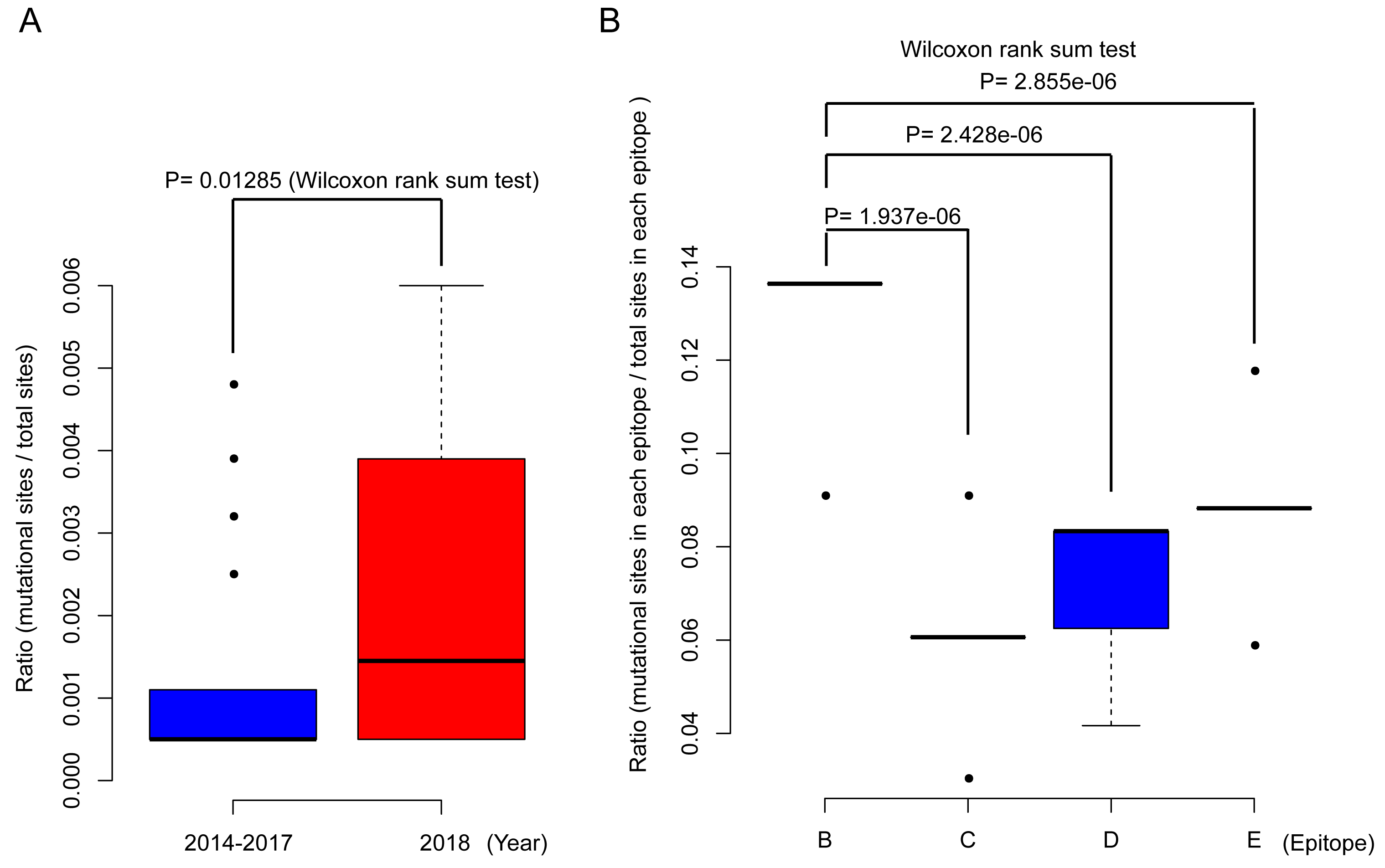

Supplement: S2 Fig — (A) Comparative analyses of accumulated variations in HA between 2018 and other year strains. (B) Comparative analyses of mutations under different epitopes for HA in 2018 strains. The significant level was decided by Wilcoxon rank sum test (P<0.05). (TIF) [file pone.0234869.s009.tif]

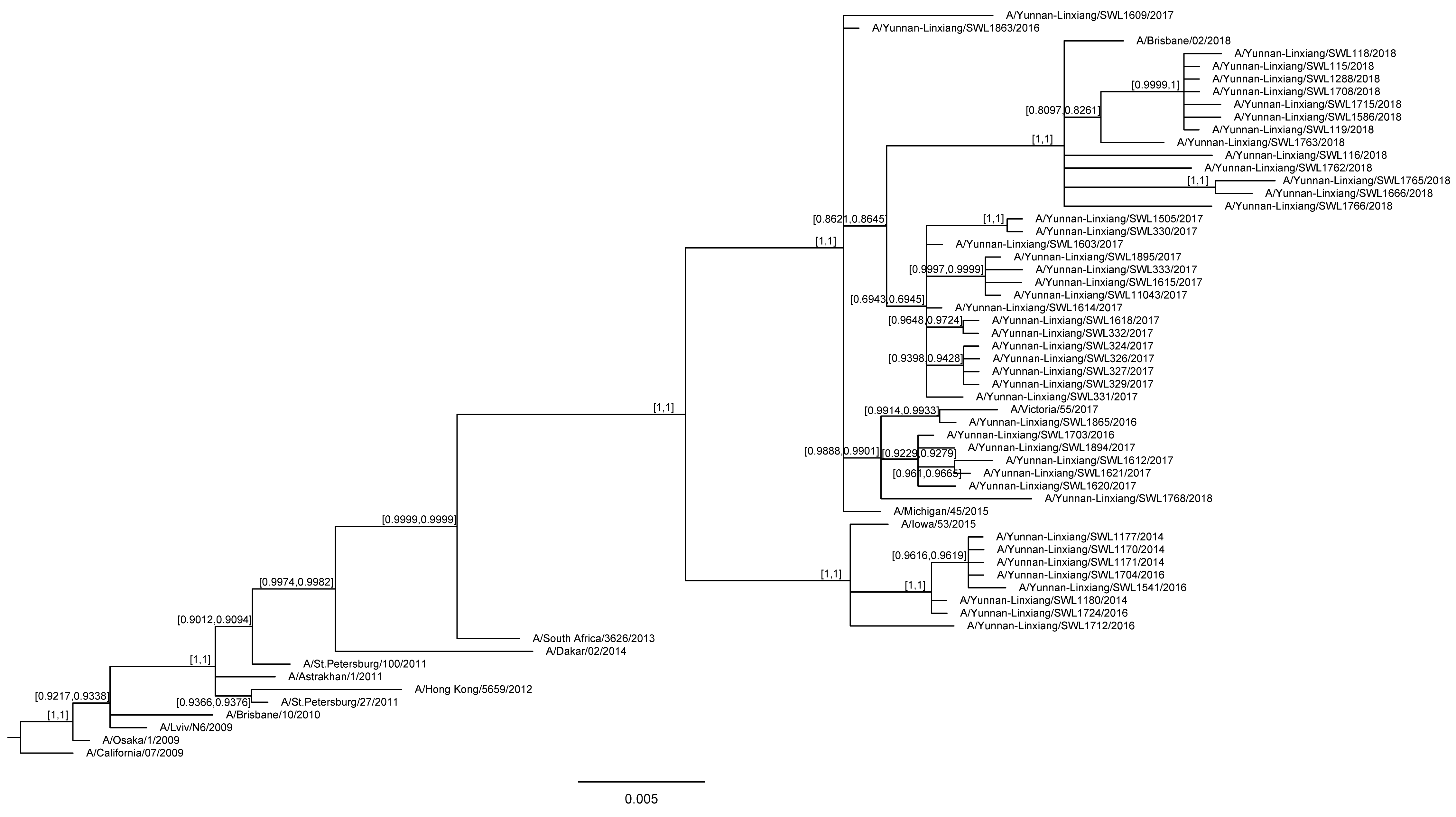

Supplement: S3 Fig — Each nodal number in square brackets represented a Bayesian posterior probability (BPP) range. The ruler value (0.005) represented genetic distance. (TIF) [file pone.0234869.s010.tif]

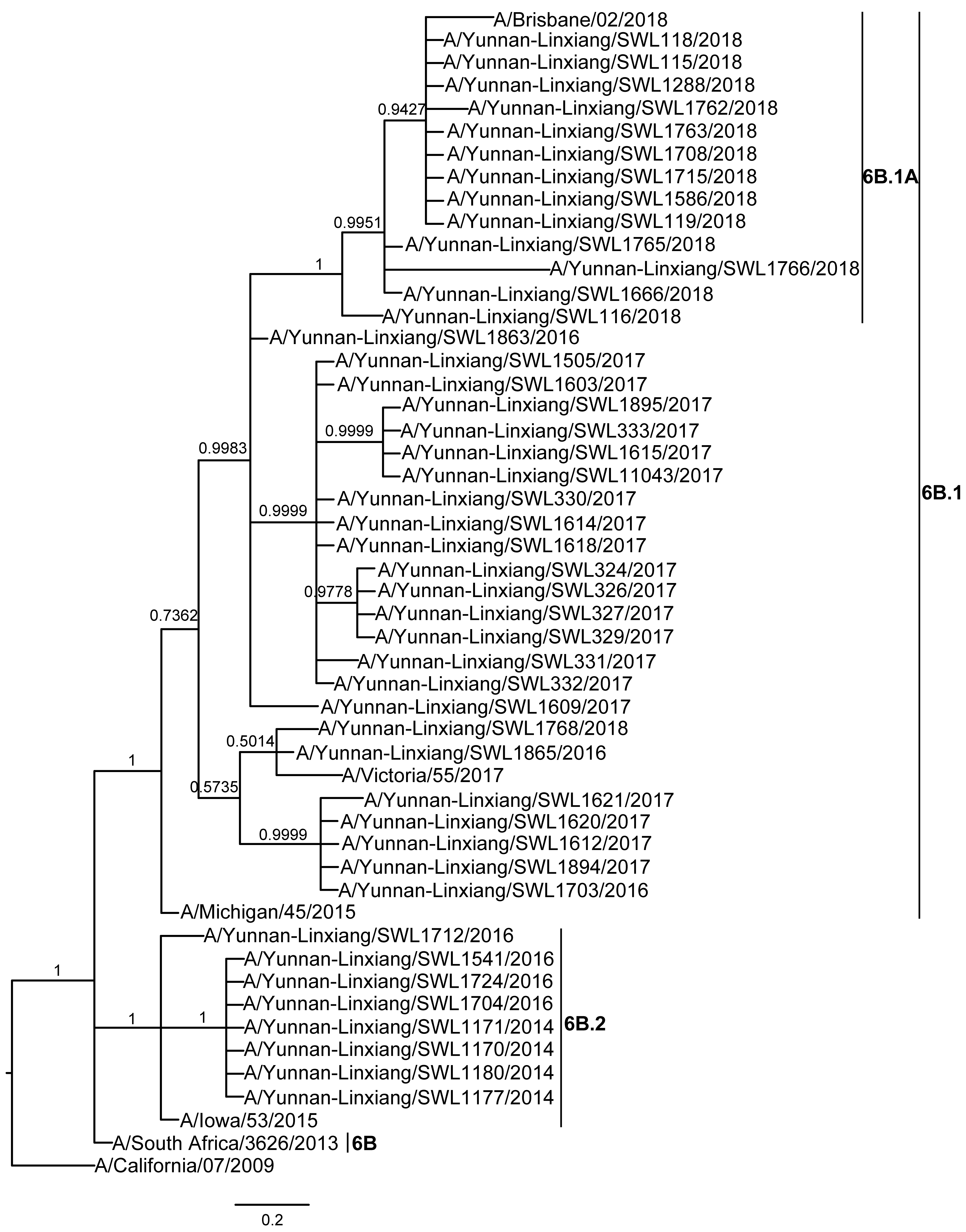

Supplement: S4 Fig — 6B~6B.1A indicated branch Numbers of clades. Each nodal number in phylogeny exhibited a Bayesian posterior probability (BPP). The ruler value (0.2) represented genetic distance. (TIF) [file pone.0234869.s011.tif]

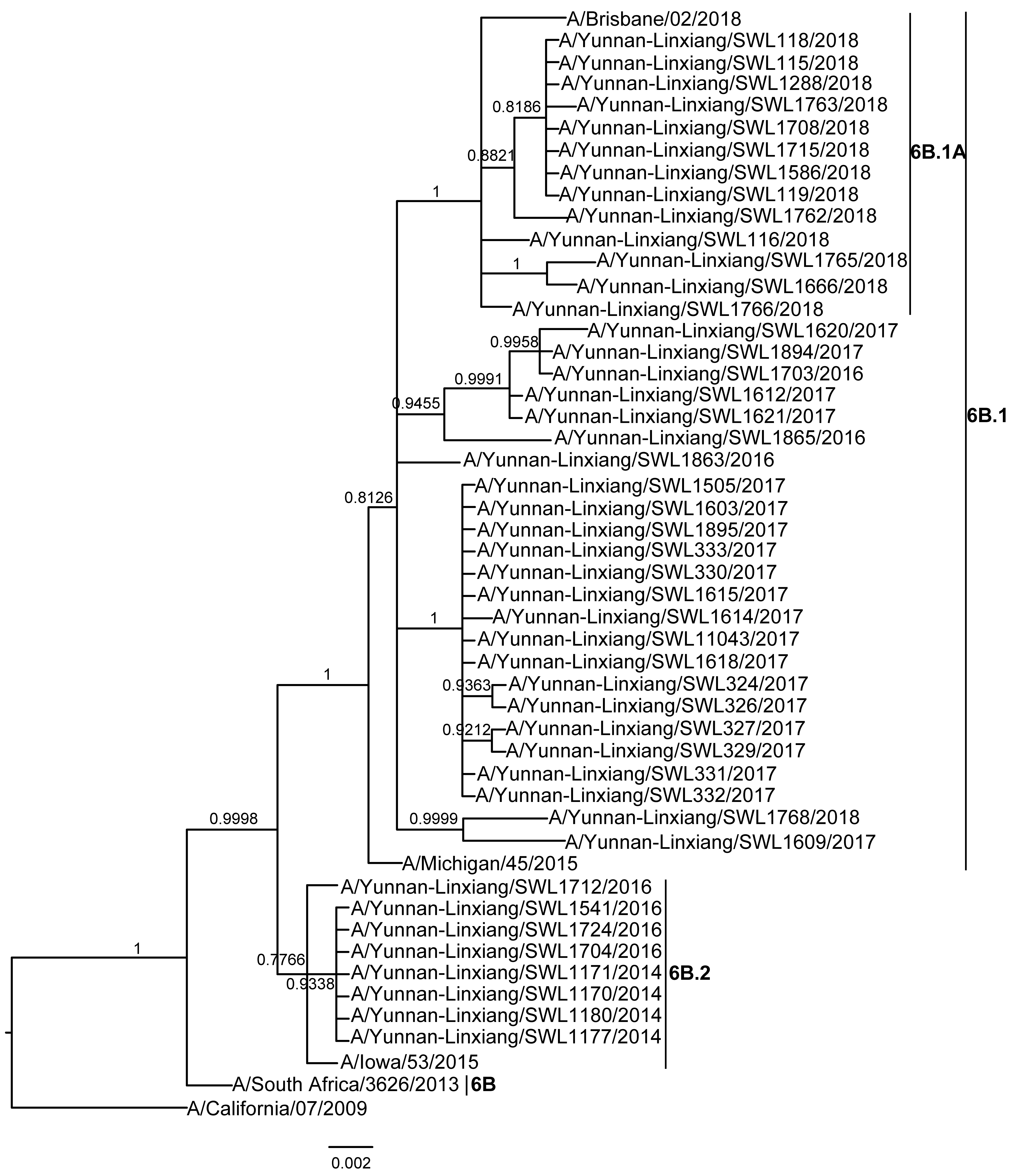

Supplement: S5 Fig — 6B~6B.1A indicated branch Numbers of clades. Each nodal number in phylogeny exhibited a Bayesian posterior probability (BPP). The ruler value (0.002) represented genetic distance. (TIF) [file pone.0234869.s012.tif]

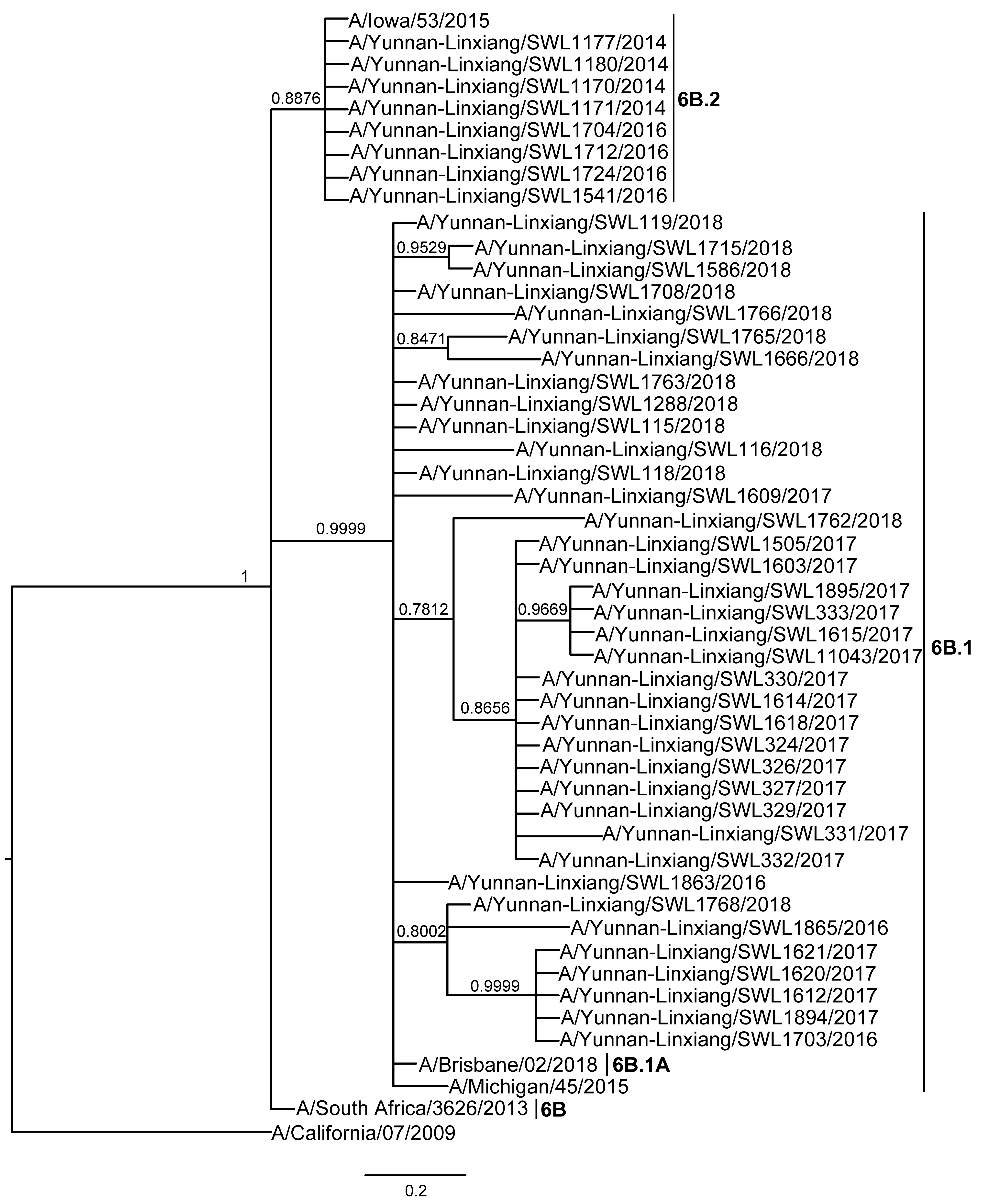

Supplement: S6 Fig — 6B~6B.1A indicated branch Numbers of clades. Each nodal number in phylogeny exhibited a Bayesian posterior probability (BPP). The ruler value (0.2) represented genetic distance. (TIF) [file pone.0234869.s013.tif]

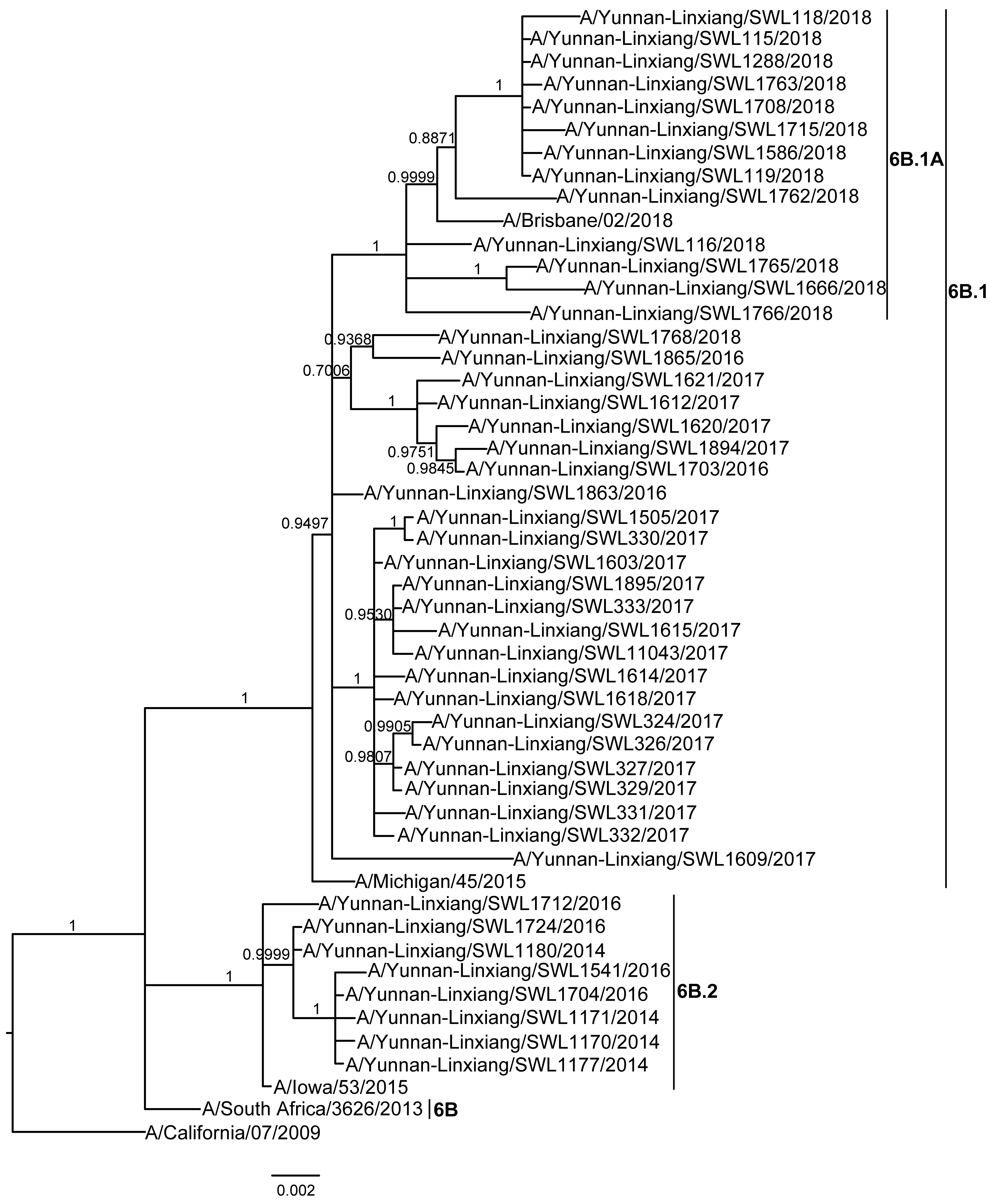

Supplement: S7 Fig — 6B~6B.1A indicated branch Numbers of clades. Each nodal number in phylogeny exhibited a Bayesian posterior probability (BPP). The ruler value (0.002) represented genetic distance. (TIF) [file pone.0234869.s014.tif]

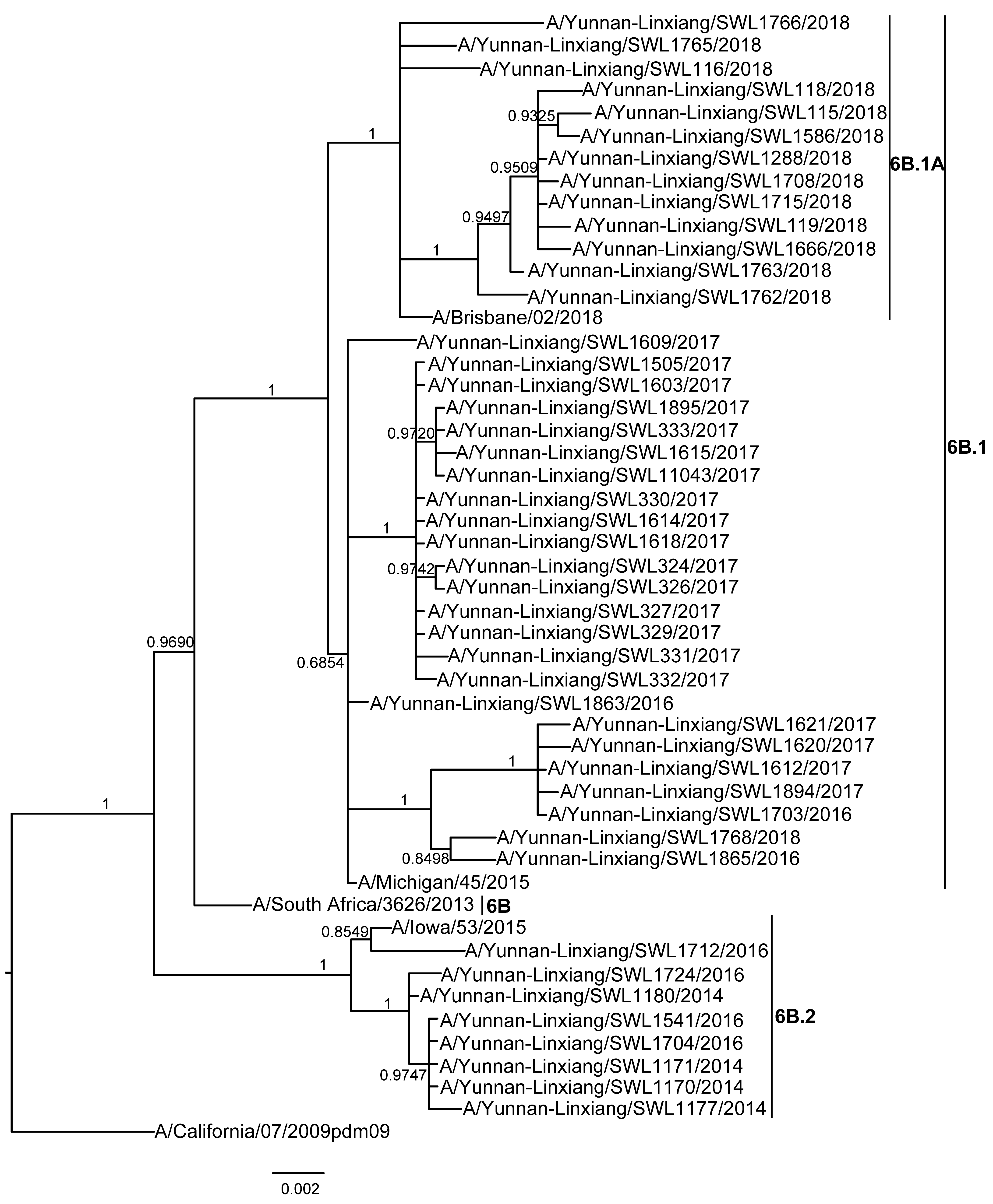

Supplement: S8 Fig — 6B~6B.1A indicated branch Numbers of clades. Each nodal number in phylogeny exhibited a Bayesian posterior probability (BPP). The ruler value (0.002) represented genetic distance. (TIF) [file pone.0234869.s015.tif]

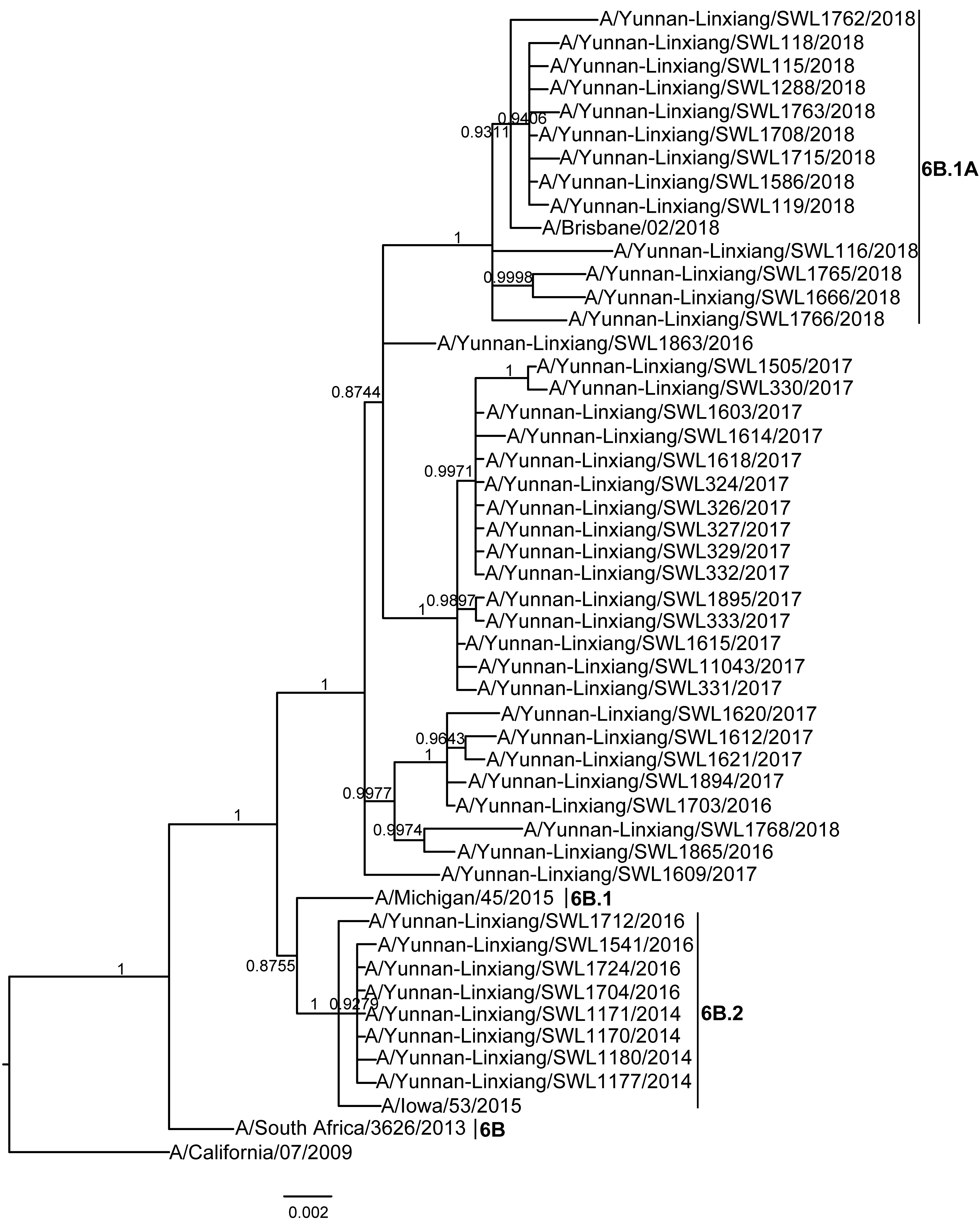

Supplement: S9 Fig — 6B~6B.1A indicated branch Numbers of clades. Each nodal number in phylogeny exhibited a Bayesian posterior probability (BPP). The ruler value (0.002) represented genetic distance. (TIF) [file pone.0234869.s016.tif]
