# Supplementary material for: Whole-genome sequencing reveals origin and evolution of influenza A(H1N1)pdm09 viruses in Lincang, China, from 2014 to 2018
Source: PLoS One. 2020 Jun 24;15(6):e0234869. doi: 10.1371/journal.pone.0234869 (PMC7314029; doi:10.1371/journal.pone.0234869)
Supplement: Attachment — Submitted filename: Response_to_reviewers_ZML_20200601.doc [file pone.0234869.s018.doc]

**Response to reviewers**

Reviewers' comments:

Reviewer's Responses to Questions

Comments to the Author

1. If the authors have adequately addressed your comments raised in a previous round of review and you feel that this manuscript is now acceptable for publication, you may indicate that here to bypass the “Comments to the Author” section, enter your conflict of interest statement in the “Confidential to Editor” section, and submit your "Accept" recommendation.

Reviewer #1: (No Response)

2. Is the manuscript technically sound, and do the data support the conclusions?

The manuscript must describe a technically sound piece of scientific research with data that supports the conclusions. Experiments must have been conducted rigorously, with appropriate controls, replication, and sample sizes. The conclusions must be drawn appropriately based on the data presented.

Reviewer #1: Partly

**Reply: According to suggestions from reviewer#1, we have fully corrected this issue in our revised manuscript.**

3. Has the statistical analysis been performed appropriately and rigorously?

Reviewer #1: Yes

4. Have the authors made all data underlying the findings in their manuscript fully available?

The PLOS Data policy requires authors to make all data underlying the findings described in their manuscript fully available without restriction, with rare exception (please refer to the Data Availability Statement in the manuscript PDF file). The data should be provided as part of the manuscript or its supporting information, or deposited to a public repository. For example, in addition to summary statistics, the data points behind means, medians and variance measures should be available. If there are restrictions on publicly sharing data—e.g. participant privacy or use of data from a third party—those must be specified.

Reviewer #1: Yes

5. Is the manuscript presented in an intelligible fashion and written in standard English?

PLOS ONE does not copyedit accepted manuscripts, so the language in submitted articles must be clear, correct, and unambiguous. Any typographical or grammatical errors should be corrected at revision, so please note any specific errors here.

Reviewer #1: Yes

6. Review Comments to the Author

Please use the space provided to explain your answers to the questions above. You may also include additional comments for the author, including concerns about dual publication, research ethics, or publication ethics. (Please upload your review as an attachment if it exceeds 20,000 characters)

Reviewer #1: The authors have largely addressed my comments. There is one significant issue, though. The formula used for efficacy is the one for H3N2. But the authors are studying H1N1. So, the author should use the efficacy formula from from Ref. 16 Figure 2, which is

efficacy = 0.53 - 1.19 pepitope

All results should be updated with this formula.

**Reply: We thank this reviewer to point out this issue for improving our manuscript. We really appreciate for your great professional knowledge in virology.**

**In this revision, according to your kind suggestions and your recommended reference and formula, we have updated all of our results throughout our manuscript including methods, results, and discussions. And we obtained a very similar efficacy trends with the previous formula. Therefore, all of revisions do not influence or change our conclusions.**

**Please check our revised manuscript in this version. We hope that our revisions can meet our requests sincerely.**

7. PLOS authors have the option to publish the peer review history of their article (what does this mean?). If published, this will include your full peer review and any attached files.

If you choose “no”, your identity will remain anonymous but your review may still be made public.

Do you want your identity to be public for this peer review? For information about this choice, including consent withdrawal, please see our Privacy Policy.

Reviewer #1: No

[NOTE: If reviewer comments were submitted as an attachment file, they will be attached to this email and accessible via the submission site. Please log into your account, locate the manuscript record, and check for the action link "View Attachments". If this link does not appear, there are no attachment files to be viewed.]

While revising your submission, please upload your figure files to the Preflight Analysis and Conversion Engine (PACE) digital diagnostic tool, https://pacev2.apexcovantage.com/. PACE helps ensure that figures meet PLOS requirements. To use PACE, you must first register as a user. Registration is free. Then, login and navigate to the UPLOAD tab, where you will find detailed instructions on how to use the tool. If you encounter any issues or have any questions when using PACE, please email us at figures@plos.org. Please note that Supporting Information files do not need this step.
